# “Nanofiltration” Enabled by Super-Absorbent Polymer Beads for Concentrating Microorganisms in Water Samples

**DOI:** 10.1038/srep20516

**Published:** 2016-02-15

**Authors:** Xing Xie, Janina Bahnemann, Siwen Wang, Yang Yang, Michael R. Hoffmann

**Affiliations:** 1 Linde+Robinson Laboratories, California Institute of Technology, Pasadena, California 91125, United States

## Abstract

Detection and quantification of pathogens in water is critical for the protection of human health and for drinking water safety and security. When the pathogen concentrations are low, large sample volumes (several liters) are needed to achieve reliable quantitative results. However, most microbial identification methods utilize relatively small sample volumes. As a consequence, a concentration step is often required to detect pathogens in natural waters. Herein, we introduce a novel water sample concentration method based on superabsorbent polymer (SAP) beads. When SAP beads swell with water, small molecules can be sorbed within the beads, but larger particles are excluded and, thus, concentrated in the residual non-sorbed water. To illustrate this approach, millimeter-sized poly(acrylamide-co-itaconic acid) (P(AM-co-IA)) beads are synthesized and successfully applied to concentrate water samples containing two model microorganisms: *Escherichia coli* and bacteriophage MS2. Experimental results indicate that the size of the water channel within water swollen P(AM-co-IA) hydrogel beads is on the order of several nanometers. The millimeter size coupled with a negative surface charge of the beads are shown to be critical in order to achieve high levels of concentration. This new concentration procedure is very fast, effective, scalable, and low-cost with no need for complex instrumentation.

Water-borne diseases are a major contributor to lower life-expectancy in the developing world^1^,^2^. A number of well-known pathogenic microorganisms (e.g., *Ascaris, Giardia, Vibrio cholera, Poliovirus*) often lead to serious health problems that compromise the quality of life in many developing countries^3^,^4^,^5^. Many pathogens have very low infectious dose levels (e.g., *Cryptosporidium parvum*, 10–30 oocysts; *Shigella*, <10 cells; *Mycobacterium tuberculosis*, <10 cells; *Escherichia. coli* (O157:H7), 2–2000 cells; and *Ebolavirus*, 1–10 cells)^6^,^7^,^8^,^9^. Thus, the ability to rapidly quantify these and other pathogens in water with a low detection limit is critically important. Although many experimental techniques are able to recognize single cells in water, sampling volumes are often limited^10^,^11^. For traditional plate-culturing methods, 100 μL or less of a water sample is spread onto a plate of nutrient media^12^. Molecular microbiology-based techniques, such as quantitative real-time polymerase chain reaction (PCR), often utilize microliter water samples^13^,^14^,^15^. Sampling volumes for microbial testing are significantly less than the suggested daily water intake for human (>2 L)^16^. Given the very low sample volumes, the measurement of specific pathogens may yield negative results, and thus the safety of the water for human consumption may be compromised. Therefore, concentration procedures during sampling or subsequently embedded into the identification technique are necessary when analyzing water samples containing low concentrations of potentially pathogenic microorganisms.

The most commonly used concentration techniques are based on various filtration methods^17^,^18^,^19^,^20^. In general, specific target organisms are either adsorbed, collected, or excluded by porous filters, while water is forced through the filter due to gravity or pressure difference. Sometimes, an eluent solution is utilized after filtration to wash the target organisms off the filter to obtain the concentrated sample. Filtration methods can concentrate water samples with large volumes (up to hundreds of liters)^21^,^22^. However, the time required for the concentration process is long and increases significantly with an increase in sample volume. In addition, application of high pressure or use of a vacuum is often required to accelerate the filtration process. The target organisms can also be separated from a water sample by using coagulation, sedimentation, or centrifugation, and then re-suspension in a liquid of less volume^17^,^23^. Other concentration techniques include evaporation, immunomagnetic separation (IMS), dielectrophoresis, and ion concentration polarization (ICP)^17^,^24^,^25^,^26^,^27^,^28^. However, the handling capacities of these methods are normally less than 1.0 L. In addition, these techniques require some common laboratory instruments, such as centrifuges, evaporators, incubators, or electrochemical workstations. These concentration technologies discussed above can be applied in a laboratory, but none of them are suitable for use in the field in developing countries, where water quality and security issues are most severe and where regular measurement of potentially pathogenic microorganisms in water samples is most needed. It is therefore of great desire to develop a fast and effective concentration technology that can concentrate microorganisms from large volumes (at least several liters) of water samples at a low cost and with minimal instrumental requirement.

Superabsorbent polymers (SAPs) can absorb and retain large amounts of liquid up to 1000 times of their own weight^29^. They become hydrogels during water sorption. Over the past 50 years, SAPs have been used in hygienic products, for protecting and sealing materials, by the agriculture and food industries, in pharmaceutical and biomedical applications, and in energy recovery or storage devices^29^,^30^. Herein, we introduce a new water sample concentration technology based on using SAP beads. As shown in Fig. 1, the new concentration procedure using SAP beads is very simple and scalable: the specially synthesized SAP beads are added into the water sample that requires concentration; the SAP beads swell with water and become hydrogel beads. During the sorption of water, some very small molecular weight molecules can be intercalated into the beads. However, particles or microbes larger than several nanometers in characteristic size are excluded and concentrated in the residual non-sorbed water. The hydrogel beads can be easily isolated from the collection vessel and reused after drying. The water sample concentration procedure is very fast, effective, scalable, low-cost, and requires no complex instruments.

## Results and Discussion

**Figure 2:**
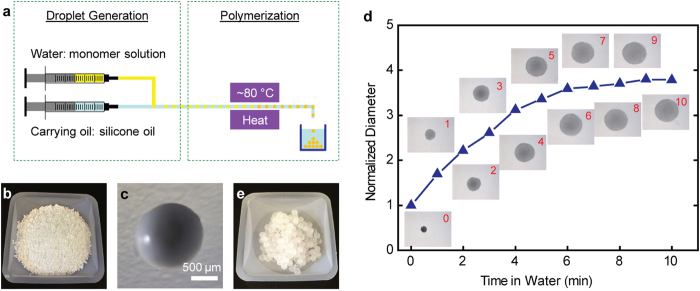
Fabrication and characterization of millimeter-sized poly(acrylamide-co-itaconic acid) beads. (**a**) Schematic of the fabrication process of the P(AM-co-IA) beads using a millifluidic system. (**b**) Picture of the P(AM-co-IA) beads as prepared. (**c**) Optical microscope image of one P(AM-co-IA) bead as prepared. (**d**) Size change of the P(AM-co-IA) beads when soaking in deionized water. The numbers at the top-right corner of the inset images indicate the soaking time. (**e**) Picture of the fully swollen P(AM-co-IA) hydrogel beads.

In order to demonstrate the concentration procedure using SAP beads for water samples, millimeter-sized poly(acrylamide-co-itaconic acid) (P(AM-co-IA)) beads were synthesized using a millifluidic system^31^,^32^,^33^. As shown in Fig. 2a, water-in-oil droplets were generated when a water phase stream (monomer solution) and an oil phase stream (silicone oil) were combined at a T-junction. These droplets flowed through a high temperature zone (~80 °C), where the monomers inside the droplets were polymerized, forming P(AM-co-IA) beads. Afterwards, these beads were collected, washed, and dried. Figure 2b shows a picture of P(AM-co-IA) beads as prepared. The optical microscope image of the bead (Fig. 2c) confirms its round shape. The diameter of these beads is 1.27 ± 0.09 mm (Fig. S1). The shape and effective diameter of the beads are determined by the flow rates of the two streams, the inner diameters of the tubes, and the monomer concentration in the droplets: beads with smaller diameter can be prepared by using smaller tubes or reducing the monomer concentration, however, the flow rates need to be adjusted. The FTIR spectrum of our product (Fig. S2) is in agreement with that reported in the literature, confirming the successful polymerization. The water absorbency was estimated by measuring the size change of the P(AM-co-IA) beads when soaking in deionized water. As shown in Fig. 2d, the diameter of the beads increases gradually and reaches ~3.8 fold of its original value within 10 minutes. Thus, the absorbency based on the volume change is about 50. Figure 2e shows the picture of fully swollen P(AM-co-IA) hydrogel beads.

**Figure 3:**
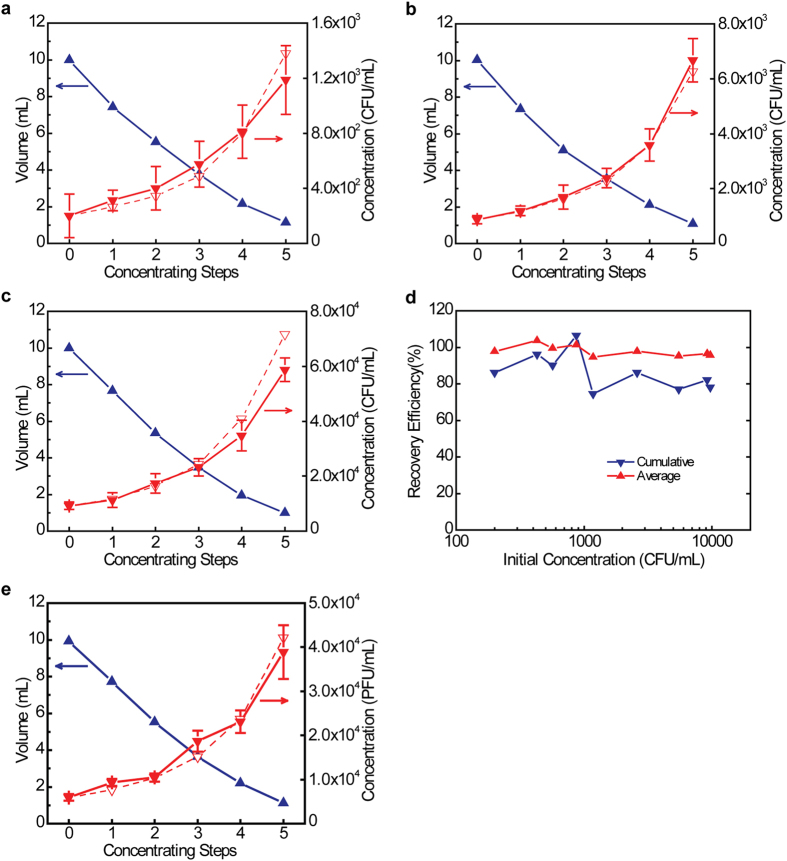
Performance of applying millimeter-sized P(AM-co-IA) beads to concentrate water samples containing microorganisms (here: *E. coli* or bacteriophage MS2). (**a**–**c**) Change in water volumes and *E. coli* concentrations during sample concentration. The initial *E. coli* concentrations are different: (**a**) ~2 × 10^2 ^CFU/mL; (**b**) ~9 × 10^2^ CFU/mL; and (**c**) ~9 × 10^3 ^CFU/mL. (**d**) Summary of the average and cumulative recovery efficiencies of the 5 concentrating steps for all concentration tests with different initial *E. coli* concentrations. (**e**) Change in water volumes and MS2 concentrations during water concentration. The initial MS2 concentration is ~6×10^3 ^PFU/mL. Dashed lines in (**a–c,e**) indicate theoretical concentrations calculated from the volume changes assuming 100% recovery during the concentration procedure.

We have used the millimeter-sized P(AM-co-IA) beads to effectively concentrate two model microorganisms, *E. coli*, a bacterium, and bacteriophage MS2, a virus, in water samples. Test water samples (10 mL) were prepared with initial *E. coli* concentrations ranging from ~2×10^2 ^CFU/mL to ~1×10^4 ^CFU/mL. Testing lower initial cell concentrations was limited by the plate-culturing method used to determine *E. coli* concentrations: the number of colonies on the plates are not sufficient to provide reliable results for quantification when *E. coli* concentrations are lower than 2 × 10^2 ^CFU/mL. The same water samples subsequently underwent five consecutive concentrating steps. In each step, 0.03–0.05 g of the SAP beads were added to the water sample; the beads were then allowed to absorb water for 10 min, after which they were separated from the solution. Water volumes and *E. coli* concentrations were measured at the beginning (V_0_ and C_0_) and after each concentrating step (V_i_ and C_i_, i = 1–5). Results presented in Fig. 3a–c and Fig. S3 show the series of steps involved in the concentration process. For example, *E. coli* concentrations increase gradually as the water volume decreases step by step as the residual volume of water approaches 1 mL. The dashed lines indicate the theoretical *E. coli* concentrations, which are calculated from the volume changes assuming no *E. coli* loss during concentration procedure. Measured concentrations closely follow the theoretical values. Recovery efficiency for each concentrating step is calculated by η_i_ = (V_i_ × C_i_)/(V_i−1_ × C_i−1_), i = 1–5. Table S1 summarizes the recovery efficiencies of all 5 concentrating steps for all concentration tests with different initial *E. coli* concentrations. For each concentration test, we calculated the average ((η_1_ + η_2_ + η_3_ + η_4_ + η_5_)/5) and cumulative recovery efficiencies (η_1_ × η_2_ × η_3_ × η_4_ × η_5_) of the 5 concentrating steps. As shown in Fig. 3d, the average recovery efficiencies are 95–104% (with a mean value of 98 ± 3%), while the cumulative recovery efficiencies are 75–106% (with a mean value of 86 ± 10%). Concentration tests using bacteriophage MS2 resulted in a similar level of performance (Fig. 3e): the average and cumulative recovery efficiencies of the 5 concentrating steps are 100 ± 19% and 92%, respectively. Efficiencies higher than 100% are the result of small measurement errors in the determination of the *E. coli* and MS2 concentrations. The experimental results demonstrate a high efficiency of the P(AM-co-IA) beads for concentrating microorganisms in water samples.

In these water sample concentration experiments, the concentration degree, namely the ratio of the sample volumes before and after concentration (V_i_/V_i−1_), of each concentration step was kept in the range of 1.3–2.1 so that the swollen SAP beads could still be suspended at the end of each concentrating step. To study the effect of the concentration degree on the recovery efficiency, water samples (10 mL) containing *E. coli* were concentrated to different volumes (2.5, 1.8, 1.6, and 0.93 mL, respectively) by single-step treatments (Fig. S4). When a 10 mL water sample was concentrated to about 2.5 mL with a concentration degree of ~4, a recovery efficiency of 88% was achieved. Increasing the concentration degrees gradually to ~10 resulted in proportional decrease of recovery efficiencies to 38%. The decline of recovery efficiency was probably caused by more *E. coli* attaching to the surface of the SAP beads: when the volume of the remaining water dropped to a certain level that is not sufficient to suspend the swollen SAP beads, the mixing condition of the system became very poor. Thus, local regions could be almost dry, forcing *E. coli* to stick on the surface of the beads. Regarding practical applications in the future, we need to consider the trade-off between concentration degree and recovery efficiency. According to the preliminary results, keeping the concentration degree of a single concentrating step less than 4 should maintain a high recovery efficiency (~90%). However, more systematic study is needed.

The superior concentrating performance of this technique depends on meeting the precondition that the target organisms (e.g., *E. coli*, bacteriophage MS2) cannot enter the SAP hydrogel beads. When the SAP beads swell with water and become hydrogel beads, the entangled polymer chains relax and form a hydrogen bonded network with the intercalated water^34^. Upon swelling, the crosslinked polymer chains form a framework with water channels interconnected inside. The relative size of the resulting water channels is a critical parameter. Only the target species with larger sizes than the swollen water channels can be effectively excluded and thus concentrated. Smaller target species may diffuse through the water channels into the hydrogel beads, resulting in relatively poor degree of concentration. This critical channel size is determined primarily by the crosslinking degree of the polymer^35^. An increase in the degree of crosslinking should effectively reduce the size of the water channels resulting in the exclusion of smaller target species. However, the water absorbency will also decrease accordingly with decreasing water channel dimensions; thus, more SAP beads may be required in order to concentrate the same amount of water.

**Figure 4:**
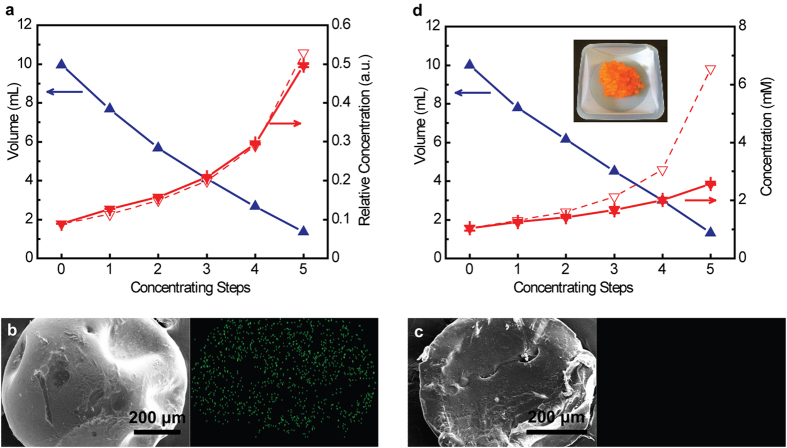
Estimation of the water channel size within the P(AM-co-IA) hydrogel beads. (**a**) Performance of applying millimeter-sized P(AM-co-IA) beads to concentrate water samples containing 5 nm gold nanoparticles (Au NPs). (**b**,**c**) Scanning electron microscope (SEM) images with energy dispersive spectrometry (EDS) elementary analysis of the outer surface (**b**) and cross-section (**c**) of the beads after drying with water containing Au NPs. Left: SEM images; Right: corresponding Au signal mapping by EDS. (**d**) Performance of applying millimeter-sized P(AM-co-IA) beads to concentrate water samples containing methyl orange (MO). Inset: P(AM-co-IA) hydrogel beads after use and intercalated by MO. Dashed lines in (**a**,**d)** indicate theoretical concentrations calculated from the volume changes assuming 100% recovery during the concentration procedure.

In order to investigate the critical channel size experimentally, P(AM-co-IA) beads were used to concentrate water samples containing gold nanoparticles (Au NPs, 5 nm) or methyl orange (MO), a common dye with an average molecule size of 2.6 nm^36^. The tests using Au NPs resulted in a high level of concentration as shown in Fig. 4a. In this case, the average and cumulative recovery efficiencies of the 5 concentrating steps are 99 ± 8% and 94%, respectively. When a water sample containing Au NPs was dried with some P(AM-co-IA) beads in it, the Au NPs only attached to the outer surface of the beads without entering the interior of the beads. SEM/EDS analysis clearly shows that the outer surface of the beads have a distinct Au signal (Fig. 4b), while the cross-sectional view of the beads does not have a Au signal (Fig. 4c). These results indicate that the size of the water channels is smaller than 5 nm. Figure 4d shows the result of the concentration test using MO. When the water sample was concentrated from 10 mL to ~1 mL, the concentration of MO increased, but much less than expected (dashed line). The cumulative recovery efficiency is only 39%. In addition, the hydrogel beads swollen with water have an obvious orange color (Fig. 4d, inset). These results indicate the intercalation of MO molecules into the hydrogel beads and suggest that the critical channel size is larger than 2.6 nm. Although it may be inappropriate to combine the results with different concentrating species directly, our experimental results indicate that the critical channel size should be about several nanometers. Therefore, the concentration procedure described herein is more appropriately described as a “nanofiltration” process with the filtered water directly absorbed by the hydrogel beads. This explains the high concentrating performance with *E. coli* and bacteriophage MS2, whose sizes are about 1–2 μm and 27.5 nm, respectively^12^,^37^.

Two other characteristics of the P(AM-co-IA) beads are important for achieving a high recovery efficiency of concentration. One important parameter is the diameter of the beads, which is designed to be around 1.0 mm. Bigger SAP beads have smaller specific surface areas and thus lower rates of water absorption. It takes several minutes for the millimeter-sized P(AM-co-IA) beads to reach their water absorption capacity. This time scale is acceptable for practical applications. Smaller SAP beads have larger specific surface areas, thus higher water absorption rates. However, larger surface areas and higher absorption rates may also increase the adsorption of concentrating objects onto the beads. To support our hypothesis, P(AM-co-IA) beads with diameter around 100 μm were synthesized via inverse suspension polymerization method (Fig. S5a–c). The FTIR spectrum of these smaller beads is almost identical to the millimeter-sized beads, indicating the same polymer structure (Fig. S5d). When added into water, the smaller beads swell and reach stable sizes within several seconds. Figure S6 shows the results of using them to concentrate a water sample containing *E. coli*. The average recovery efficiency of 5 concentrating steps is 84 ± 12%, and the cumulative efficiency is less than 40%, significantly lower than that achieved with previous millimeter-sized beads. In addition to having a decreased concentrating performance, the smaller beads are more difficult to separate from water after use. Therefore, a moderate bead size is preferred to balance the trade-off between concentration performance and treatment time, and further optimization is needed.

The other important characteristic is the surface charge of the hydrogel beads. At circum-neutral pH, P(AM-co-IA) should be negatively charged, because of the carboxylate groups introduced by the co-polymerization of IA^38^,^39^. The negative surface charge of the beads exerts an electrostatic repulsion toward negatively-charged microorganisms during the concentration procedure at circum-neutral pH^40^,^41^. In order to investigate the roll of the negative surface charge, PAM beads were synthesized using the same millifluidic method. The diameter of these beads is 1.12 ± 0.03 mm (Fig. S7a and b). The absence of peak at ~1550 cm^−1^ for carboxylate groups in the FTIR spectrum indicates no IA in the PMA beads (Fig. S7c)^42^. Without IA component, PAM should be almost neutral at pH around 7^43^,^44^. The PAM beads were tested for sample concentration. As the results shown in Fig. S8, the *E. coli* concentrations in the concentrate are much less than the expectation (dashed line). The cumulative recovery efficiency is only 19%. This result highlights the importance of the negative surface charge of the beads when concentrating microorganisms.

In addition to a high level of concentration over very short periods of time, our new approach to water sample concentration has several advantages. First, the process has a very low-cost. The SAPs beads are made from inexpensive chemical components, which have been used in personal, disposable hygienic products, such as baby diapers and sanitary napkins. In contrast, the SAP hydrogel beads can be reused for subsequent concentration applications after drying. At room temperature with natural ventilation, it only takes only 2 to 3 hours for the swollen P(AM-co-IA) hydrogel beads to lose most of the absorbed water by evaporation such that they shrink back to a size of ~1 mm (Fig. S9a). Using recycled P(AM-co-IA) beads for concentrating *E. coli* in a water sample, we were able to achieve a similar level of performance (Fig. S9b and c) compared to the newly prepared beads. Second, the operation of our technology is very simple, fast, and scalable. Little training would be required for the reliable and reproducible use by a technician. The overall time required for the concentration process is on the order of only several minutes and it is independent to the volume of water sample treated. Larger sample volumes would only require using additional SAP beads. The third advantage is that concentrating water samples with SAP beads does not involve complex instrumentation and the energy consumption is negligible. With these important features, our new approach to water sample concentration shows great promise for practical applications in microbial sampling and quantification in the field or for use in developing areas of the world where standard lab facilities are not readily available.

## Methods

### Materials

Acrylamide (AM), itaconic acid (IA), potassium persulfate (KPS), *N*,*N*’-methylenebisacrylamide (Bis-A), silicone oil, cyclohexane, chloroform, sorbitan monooleate (Span-80), polyoxyethylene-60-sorbitan monooleate (Tween-60), and Gold nanoparticles (Au NPs) were purchased from Sigma-Aldrich. Luria-Bertani broth (LBB), Luria-Bertani agar (LBA), tryptic soy broth (TSB), and tryptic soy agar (TSA) were purchased from Fisher Scientific. Methyl orange (MO) was purchased from Baker Analyzed ^TM^. All chemicals are used as received. Microbial strains were purchased from ATCC.

### Preparation and characterization of SAP beads

#### Preparation of millimeter-sized P(AM-co-IA) beads

Millimeter-sized P(AM-co-IA) beads were synthesized using a millifluidic system. First, water-in-oil droplets were generated when a water phase stream and an oil phase stream were combined at a T-junction. The water phase includes 180 g/L AM, 20 g/L IA, 2.6 g/L KPS, and 4.0 g/L Bis-A^45^. The oil phase is silicone oil. The inner diameter of the silicone tubing is 1/16 inch. Two independent high-precision syringe pumps (Cole-Parmer) were applied to inject the streams. The flow rate of water and oil were 0.2 mL/min and 0.3 mL/min, respectively. These flow rates had been adjusted and optimized to achieve water-in-oil droplets with size similar to the inner diameter of the silicone tubing and with aspect ratio close to one. Second, the water-in-oil droplets flowed through a water bath with a temperature of ~80 °C, which allows the polymerization of the monomers inside the droplets. Last, the beads produced were subsequently separated from the carrying oil by a strainer, washed with ethanol, and dried under vacuum at 40 °C overnight. The resulted dry SAP beads are smaller than the droplets due to the loss of original water content.

#### Preparation of millimeter-sized PAM beads

Millimeter-sized PAM beads were prepared by the same millifluidic method, except containing 200 g/L AM and no IA in the water phase.

#### Preparation of 100-micrometer-sized P(AM-co-IA) beads

P(AM-co-IA) beads with smaller sizes were synthesized via an inverse suspension polymerization method. Specifically, 40 mL monomer solution as described before was charged into a 500 mL flask containing 200 mL cyclohexane and chloroform (2:1) solution with 4 g/L Span-80 and 2 g/L Tween-60. Under stirring at ~300 rpm, the mixture was kept at 50 °C for 40 min and subsequently 70 °C for 2 h. After the mixture cooled to room temperature, P(AM-co-IA) beads with diameter around 100 μm were precipitated by adding ethanol. The precipitate was washed with ethanol and dried under vacuum at 40 °C overnight.

#### Characterization of the SAP beads

The structures of the SAP beads were analyzed by Fourier transform infrared spectroscopy (FTIR, Thermo Scientific) and the sizes were measured under optical microscope (Leica M205 FA). Scanning electron microscope (SEM) images and the energy dispersive spectrometry (EDS) elementary analysis were performed by a field emission scanning electron microscope (ZEISS 1550VP) equipped with EDS system (Oxford X-Max SDD). SEM samples were coated with 5 nm carbon to improve the conductivity.

### SAP beads for water sample concentration

#### Preparation of the E. coli (ATCC 10798) stock

After incubation in LBB at 37 °C for 16–18 hours, *E. coli* cells were harvested by centrifugation at 5000 × g for 5 min and washed with deionized water for three times.

#### Preparation of bacteriophage MS2 (ATCC 15597-B1) stock

Bacteriophage MS2 was first cultured with the host *E. coli* (ATCC 15597) in TSB at 37 °C for 4–6 hours. The TSB with cells was subsequently centrifuged at 4000 × g for 5 min and the supernatant filtered through a 0.2 μm filter to remove the residual *E. coli* cells.

#### Concentration of water samples

A typical concentrating experiment was performed as follows: 1) Prepare 10 mL water sample containing specific concentrating object (e.g., *E. coli*, bacteriophage MS2, Au NPs, or MO) with specific initial concentrations. 2) Weight certain amount of SAP beads (normally 0.03–0.05 g), and add them into the water sample. 3) Shake the container gently for 10 min. 4) Transfer the remaining water into another container with pipette. 5) Take 200 μL sample for concentration measurement. 6) Repeat step 2–5 for 4 times to concentrate the water sample to about 1 mL. The volumes of the water remained after each treatment and the concentrations of the object in the initial and concentrated water samples were carefully analyzed.

#### Characterization of water samples

The *E. coli* concentrations were measured using standard spread plating techniques with five replicates for each measurement. Bacteriophage MS2 was quantified by a double agar layer method. The concentrations of Au NPs and MO were determined spectroscopically using a UV-Vis Spectrophotometer (Nanodrop 2000C, Thermo Scientific).

## Additional Information

**How to cite this article**: Xie, X. *et al.* "Nanofiltration" Enabled by Super-Absorbent Polymer Beads for Concentrating Microorganisms in Water Samples. *Sci. Rep.* **6**, 20516; doi: 10.1038/srep20516 (2016).

## Supplementary Material

Supplementary Information

## Figures and Tables

**Figure 1:**
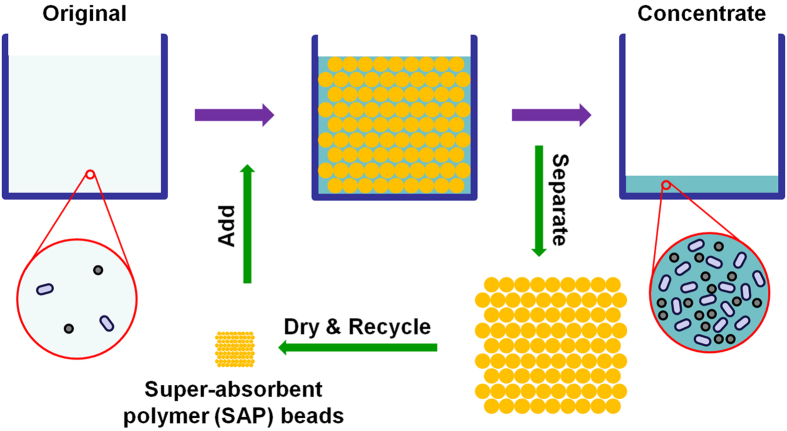
Schematic diagram illustrating the water sample concentration procedures using super-absorbent polymer (SAP) beads. The standard operation includes: (1) adding synthesized SAP beads into a water sample that requires a concentration step in order for a specific analytical procedure; (2) exposure of the beads for given amount of time for water uptake; (3) separating the swollen beads to collect the concentrate. The SAP beads can be recycled after drying.
