# Supplementary material for: “Nanofiltration” Enabled by Super-Absorbent Polymer Beads for Concentrating Microorganisms in Water Samples
Source: Sci Rep. 2016 Feb 15;6:20516. doi: 10.1038/srep20516 (PMC4753426; doi:10.1038/srep20516)
Supplement: Supplementary Information [file srep20516-s1.pdf]

Supporting Information for:

"Nanofiltration" Enabled by Super-Absorbent Polymer Beads for Concentrating Microorganisms in Water Samples

Xing Xie[1], Janina Bahnemann[1], Siwen Wang[1], Yang Yang[1], Michael R. Hoffmann[1]

[1]Linde+Robinson Laboratories, California Institute of Technology, Pasadena, California 91125, United States.

Correspondence and requests for materials should be addressed to M.R.H. (mrh@caltech.edu).

Submitted to *Scientific Reports*

Octorber 2015

Supplementary Figures

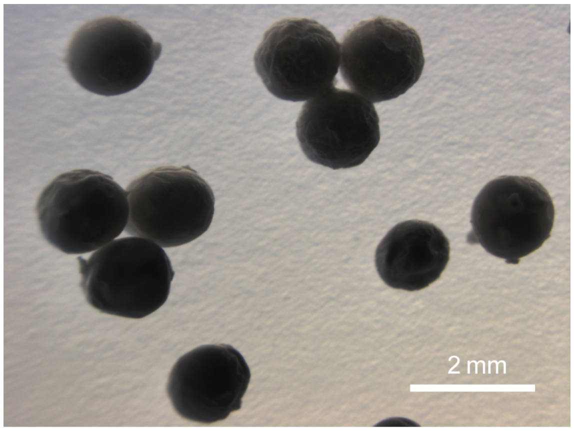

Figure S1 | Optical microscope images of P(AM-co-IA) beads as prepared.

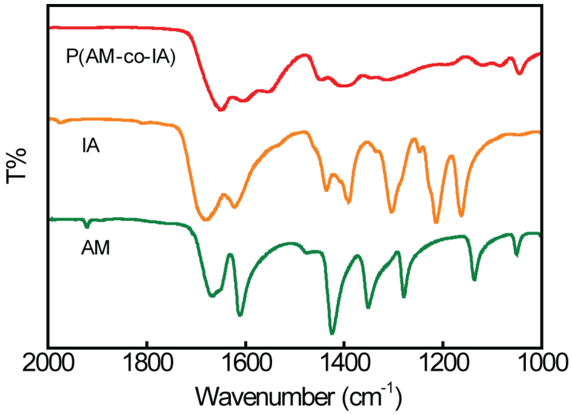

Figure S2 | FTIR spectrum of P(AM-co-IA) and the monomers AM and IA.

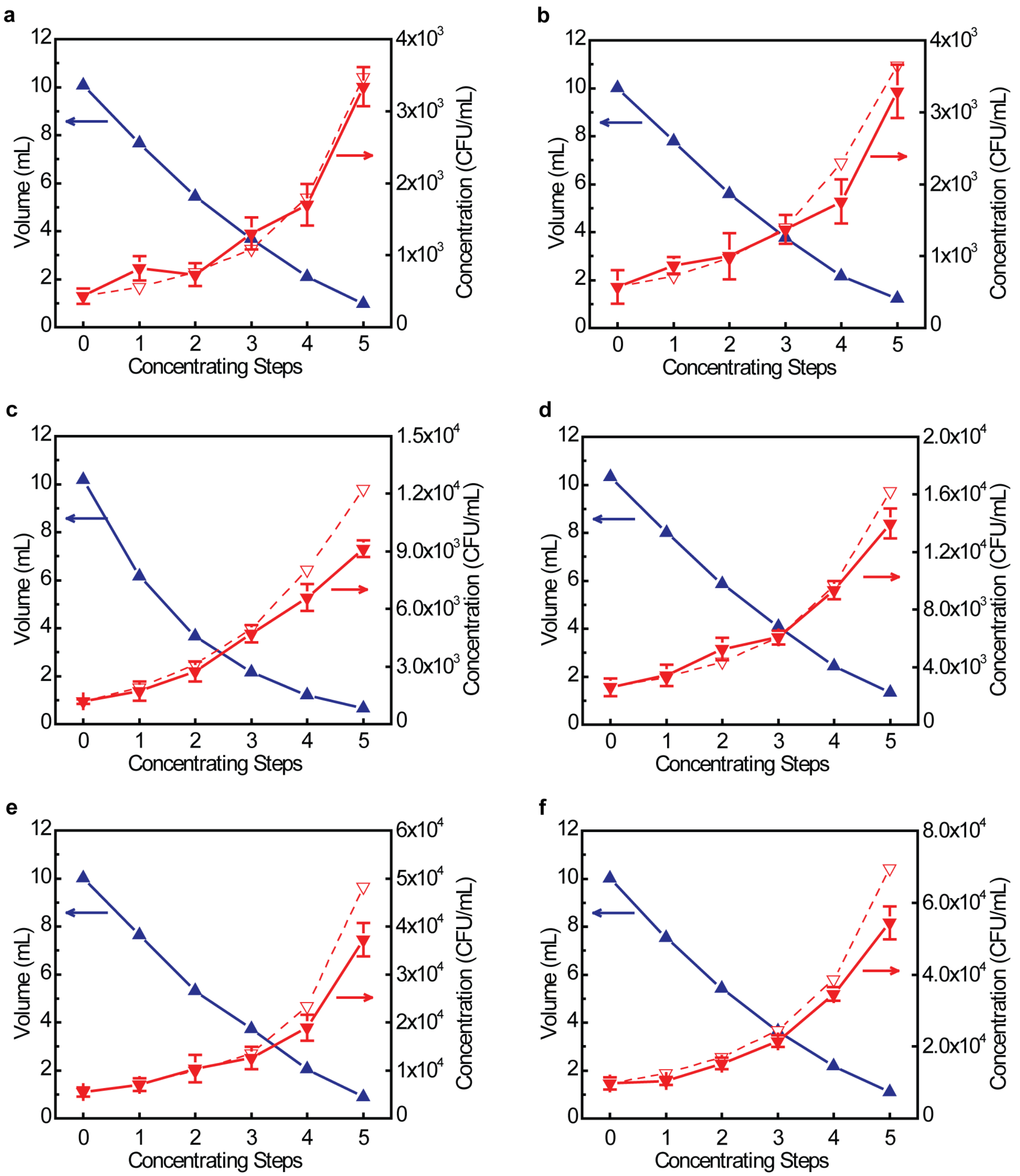

Figure S3 | Additional results of applying millimeter-sized P(AM-co-IA) beads to concentrate water samples containing *E. coli*. (**a-f**) Change in water volumes and *E. coli* concentrations during sample concentration. Initial *E. coli* concentrations are different: **a**, ~4×10^2 CFU/mL; **b**, ~6×10^2 CFU/mL; **c**, ~1×10^3 CFU/mL; **d**, ~3×10^3 CFU/mL; **e**, ~6×10^3 CFU/mL; and **f**, ~1×10^4 CFU/mL. Dashed lines in **a-f** indicate theoretical concentrations calculated from the volume changes assuming 100% recovery during the concentration procedure.

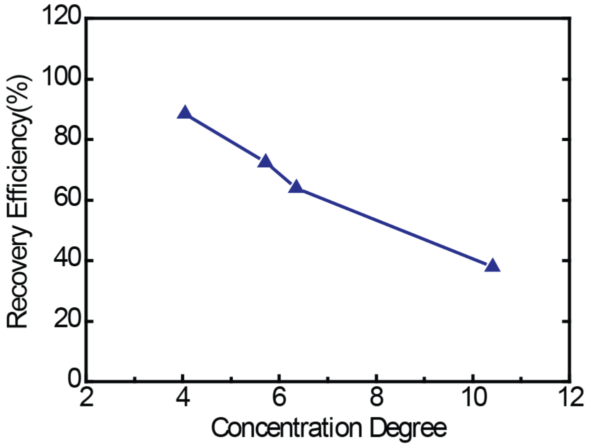

Figure S4 | Recovery efficiencies of single concentrating steps with different concentration degrees. Water samples (10 mL) containing *E. coli* were concentrated to 2.5, 1.8, 1.6, and 0.93 mL, respectively.

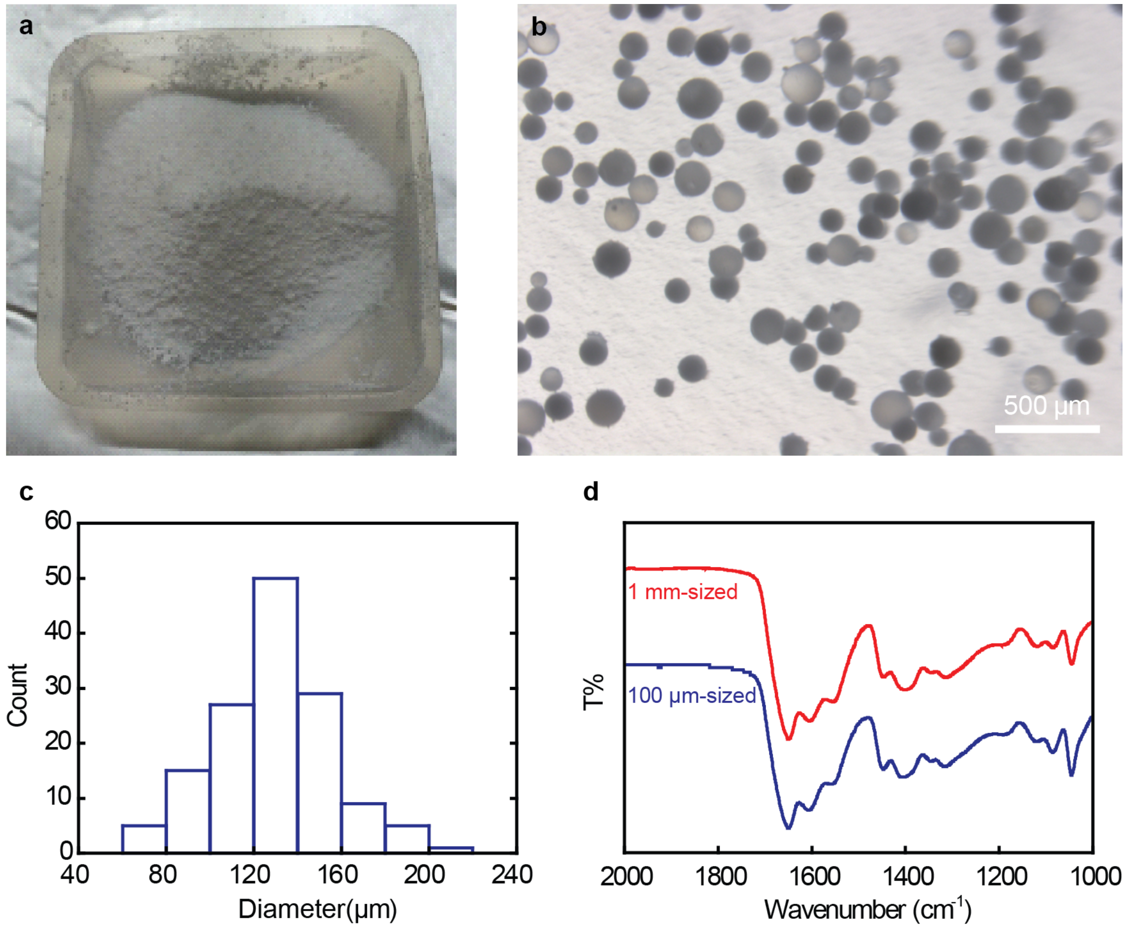

Figure S5 | Characterization of 100-micrometer-sized P(AM-co-IA) beads. (**a**) Picture of the P(AM-co-IA) beads as prepared. (**b**) Optical microscope image of P(AM-co-IA) beads as prepared. (**c**) Size distribution of over 100 beads. (**d**) FTIR spectrum of P(AM-co-IA) beads with different sizes.

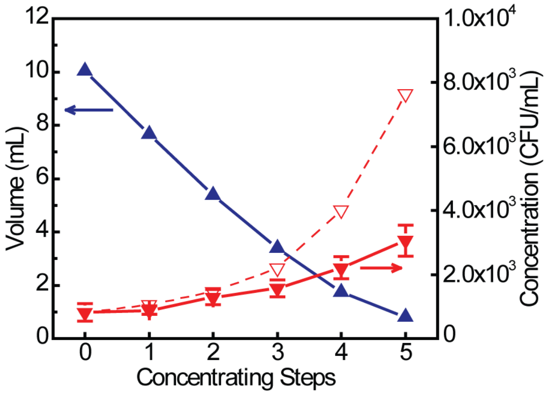

Figure S6 | Performance of applying 100-micrometer-sized P(AM-co-IA) beads to concentrate water samples containing *E. coli.* The figure shows the change in water volumes and *E. coli* concentrations during sample concentration. The initial *E. coli* concentration is ~8×10^2 CFU/mL. Dashed line indicates theoretical concentrations calculated from the volume changes assuming 100% recovery during the concentration procedure.

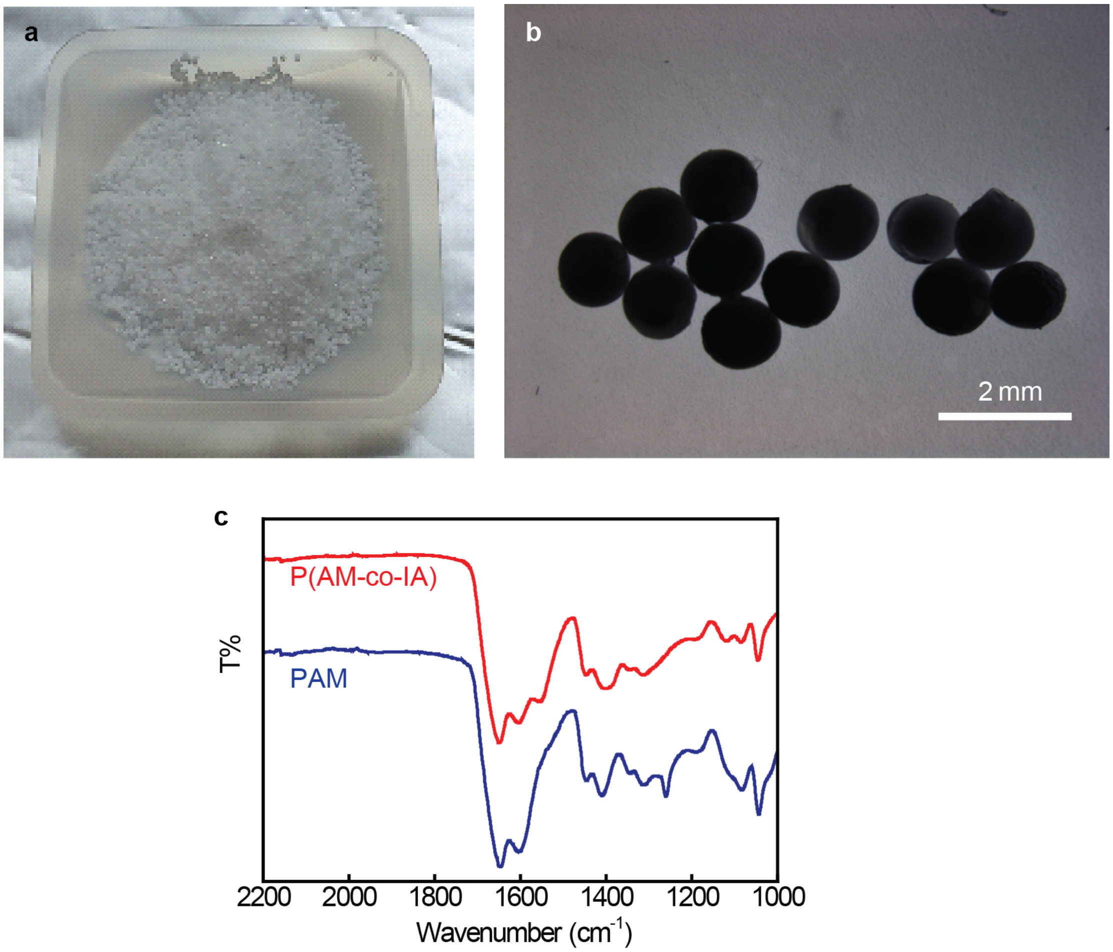

Figure S7 | Characterization of millimeter-sized PAM beads. (**a**) Picture of the PAM beads as prepared. (**b**) Optical microscope image of PAM beads as prepared. (**c**) FTIR spectrum of PAM beads and P(AM-co-IA) beads.

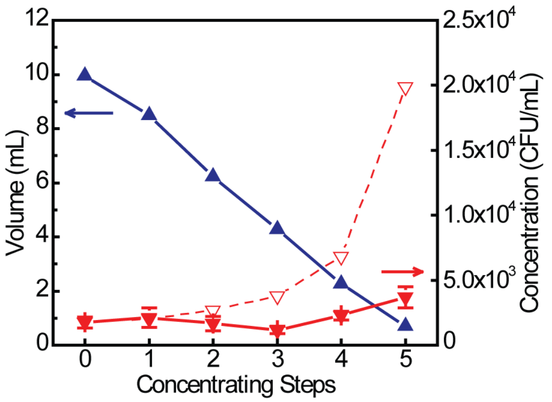

Figure S8 | Performance of applying millimeter-sized PAM beads to concentrate water samples containing *E. coli*. The figure shows the change in water volumes and *E. coli* concentrations during sample concentration. The initial *E. coli* concentration is ~2×10^3 CFU/mL. Dashed line indicates theoretical concentrations calculated from the volume changes assuming 100% recovery during the concentration procedure.

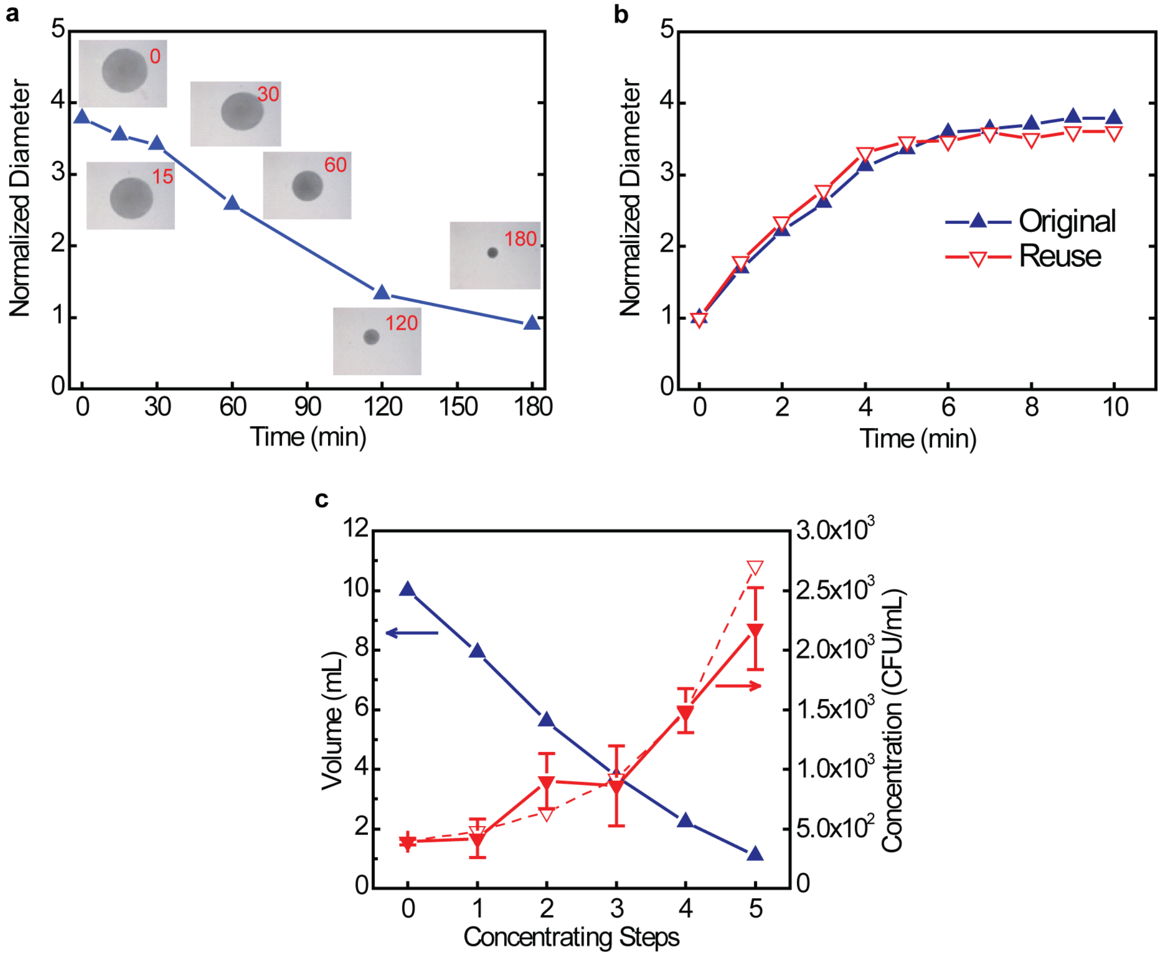

Figure S9 | Recycle of millimeter-sized P(AM-co-IA) beads. (**a**) Size change of the P(AM-co-IA) beads under room temperature and natural ventilation. The numbers at the top-right corner of the inset images indicate the drying time. (**b**) Size change of the recycled P(AM-co-IA) beads when soaking in deionized water, in comparison with the original beads. No obvious difference is observed. (**c**) Performance of using the recycled P(AM-co-IA) beads to concentrate water samples containing *E. coli*. The figure shows the change in water volumes and *E. coli* concentrations during sample concentration. Initial *E. coli* concentration is ~4×10^2 CFU/mL. Dashed line in **c** indicates theoretical concentrations calculated from the volume changes assuming 100% recovery during the concentration procedure.

Table S1 | Recovery efficiencies of the 5 concentrating steps for all concentration experiments with different initial *E. coli* concentrations

Initial Concentration (CUF/mL)	Concentrating Steps					Average	Cumulative
	1	2	3	4	5		
2.0×10^{2}	118.3	97.9	101.9	85.8	85.0	97.8	86.1
4.3×10^{2}	147.6	65.0	124.9	78.9	101.7	103.6	96.2
5.7×10^{2}	121.3	84.8	95.5	78.0	117.6	99.4	90.2
8.7×10^{2}	102.3	101.2	101.5	95.6	106.0	101.3	106.4
1.2×10^{3}	90.0	97.9	107.9	86.3	90.9	94.6	74.6
2.6×10^{3}	104.0	115.5	83.3	96.4	89.4	97.7	86.2
5.5×10^{3}	99.1	105.5	88.8	87.2	95.3	95.2	77.1
9.2×10^{3}	95.6	110.8	89.9	89.1	96.7	96.4	82.0
9.7×10^{3}	82.6	108.4	97.8	102.4	87.3	95.7	78.2
Average	NA	NA	NA	NA	NA	**98.0**	**86.3**
